# Can nitrogen supersede host identity in shaping the community composition of foliar endophytic fungi in an alpine meadow ecosystem?

**DOI:** 10.3389/fmicb.2022.895533

**Published:** 2022-08-22

**Authors:** Yiming Meng, Qi Zhang, Guoxi Shi, Yongjun Liu, Guozhen Du, Huyuan Feng

**Affiliations:** ^1^Ministry of Education Key Laboratory of Cell Activities and Stress Adaptations, School of Life Sciences, Lanzhou University, Lanzhou, China; ^2^College of Bioengineering and Biotechnology, Tianshui Normal University, Tianshui, China; ^3^Center for Grassland Microbiome, Lanzhou University, Lanzhou, China; ^4^State Key Laboratory of Grassland Agro-Ecosystems, Lanzhou University, Lanzhou, China; ^5^School of Life Sciences, Lanzhou University, Lanzhou, China; ^6^Key Laboratory of Arid and Grassland Ecology of Ministry of Education, School of Life Sciences, Lanzhou University, Lanzhou, China

**Keywords:** foliar fungal endophytes, nitrogen deposition, Qinghai–Tibet Plateau, microbial community, host-specificity

## Abstract

The availability of limiting nutrients plays a crucial role in shaping communities of endophytes. Moreover, whether fungal endophytes are host-specific remains controversial. We hypothesized that in a harsh and nitrogen (N)-deficient area, diversity and community composition of foliar endophytic fungi (FEFs) varied substantially among plots with experimentally elevated levels of macronutrients, and thus, N availability, instead of host species identity, would have a greater influence in structuring fungal communities at different scales. We also expected an important subset of taxa shared among numerous host species and N gradients to form a community-wide core microbiome. We measured the leaf functional traits and community structures of FEFs of three commonly seen species in an alpine meadow nested with a long-term N fertilization experiment. We found that host plant identity was a powerful factor driving the endophytic fungal community in leaves, even in habitats where productivity was strongly limited by nitrogen (*p* < 0.001). We also found that within the same host, nitrogen was an important driving force for the composition of the endophytic fungi community (*p* < 0.05). In addition, the leaf carbon content was the most important functional trait that limited the diversity of endophytic fungi (*p* < 0.001). Finally, we documented a distinct core microbiome shared among our three focal species and N gradients.

## Introduction

Ecosystems worldwide have been undergoing severe nitrogen (N) deposition over the last few decades (Cardinale et al., 2011; IPCC, 2014). In terrestrial ecosystems, N availability is supposed to be a primary limitation to plant growth (Vitousek and Howarth, 1991). Previous research has shown that N deposition, simulated by N fertilization experiments, increases plant growth and crop yields. However, N losses from industry and agriculture alter the plant community structure and reduce terrestrial biodiversity (Stevens et al., 2004; Bobbink et al., 2010). N deposition also results in an increase in air pollution (EPA, 2013) and soil acidification (Sullivan et al., 2013) and is hence considered a major threat to ecosystem functioning and services. While N deposition has declined in some parts of the world due to legislation, it is estimated that soil communities, ecosystem productivity, and nutrient cycling continue to be affected by historical soil nitrogen loads (Gilliam et al., 2019; Crawford et al., 2020).

Although the effect of atmospheric N deposition on plant ecology has been widely studied (Ellison and Gotelli, 2002; Scarpitta et al., 2017; Vallicrosa et al., 2022), our understanding of how N shapes the microbial community, particularly the fungal community, inside plants remains elusive. Plants do not exist as axenic organisms but are closely associated with large quantities of microorganisms. Leaves comprise one of the world's largest terrestrial habitats and provide a key location for interactions between plants and their associated microbiomes, yet we know relatively little about this association (Vacher et al., 2016). Foliar endophytic fungi (FEFs) spend all or part of their lifetime colonizing plant leaves and may increase, decrease, or show no apparent impact on host performance, as reviewed by Rodriguez et al. (2009) and Hardoim et al. (2015). Such mutualistic parasitic symbiosis depends on the host–endophyte genotype–genotype interaction, environmental conditions, and the state of the host's health (Stevens et al., 2004). However, the mechanism of this complex interactive network remains unclear. Moreover, although existing research indicates that FEFs mediate plant N uptake (Buckley et al., 2019; Christian et al., 2019), how N input affects the community structure of the FEF remains relatively unexplored. FEFs represent a ubiquitous and highly diversified fungal guild (Rodriguez et al., 2009) and a substantial and mostly under-explored microbiota critical for ecosystem functioning (Hardoim et al., 2015). Despite the ecological importance, the factors that shape FEF communities, either biotic or abiotic, are largely unknown.

Plant species shape biotic and abiotic environments in which the fungi live, and hence, different FEF species might be a function of host plant phylogeny (Apigo and Oono, 2018). However, the degree that FEFs are host-specific is controversial. Some endophytes were unique to divergent host clades in boreal and arctic sites (Higgins et al., 2007; Zhang and Yao, 2015), and they also showed a degree of host specificity in rainforest trees (Vincent et al., 2016), tropical trees (Arnold and Lutzoni, 2007), and grasses (Higgins et al., 2011, 2014). If high specificity is prevalent, this may have profound implications for plant–fungus coevolution, as plants can vary dramatically in susceptibility to their parasitic fungal companions, and plants prefer harboring mutualistic fungal companions (Clay and Schardl, 2002; Saikkonen et al., 2004; Chen et al., 2019; Razali et al., 2019). Conversely, it was also found that distantly related pine species hosted the same FEF species, indicating host species did not influence the microbial genetic structure (Oono et al., 2014). This might be explained by the fact that FEFs are often horizontally transmitted (Arnold et al., 2009; Rodriguez et al., 2009); thus, some populations may be panmictic, reflecting generalist associations with multiple host species (Dunham et al., 2013). Altogether, much evidence needs to be accumulated to understand the host specificity of FEF communities. Understanding the host specificity of FEFs will also provide critical insights into plant performance, competitive abilities among species, and the FEF dispersal strategy.

N input increases plant biomass and enhances plant growth. Changes in plant growth may further lead to variations in the plant-associated fungal community structure (Seghers et al., 2004; Larkin et al., 2012; Suryanarayanan and Shaanker, 2021). Furthermore, over different growth stages of the host plant, the plant endophytic fungal community is likely subject to dynamic changes over time (Singha et al., 2021; Sosso et al., 2021; Zheng et al., 2021). Several studies have provided some insights into how FEFs respond to high N supply. An inoculation experiment showed that endophyte (*Neotyphodium lolii*) concentration was reduced by 40% under high N supply in perennial ryegrass (*Lolium perenne*) cultivars (Rasmussen et al., 2007). Data from several sources also identified that FEFs enhanced plant N uptake and growth based on inoculation experiments (Newman et al., 2003; Knoth et al., 2013; Christian et al., 2019). However, all these conclusions were drawn based on greenhouse-based experiments with inoculation of specific species of endophytic fungi. How macronutrients, particularly N, regulate endophytic fungal communities in natural systems remains controversial. For example, Larkin et al. (2012) reported that nitrate in leaves was correlated with differences in the endophyte community, while in another study, Huang et al. (2016) found no correlation between foliar N and endophytic abundance or diversity in one host species, but a negative correlation with endophytic abundance in another species was found.

Independent of whether FEFs are highly host-specific, there still may be a subset of taxa that are shared among co-occurring host species. For example, Zhang and Yao (2015) found that 7.6% of total FEF operational taxonomic units (OTUs) were shared by all four plant species in the study of vascular plants in the high arctic zone. Thus, a very small group of fungi occurred repeatedly among plant species. Some subsets of fungi that comprise the shared OTUs (or, “core microbiome”, see the explanation from Vandenkoornhuyse et al., 2015 and Shade and Handelsman, 2012) may have traits that enhance their ability to colonize the interior of a phylogenetically diverse group of plant species, indicating their ecological competitiveness and importance. However, such investigations are very few, therefore, we cannot draw general conclusions on commonly shared FEF OTUs among plant species in different habitats.

The Qinghai–Tibet Plateau (QTP) is one of the hot spots for research, yet little is known about the endophytic fungal community and its response to global change in this region. Learning endophytic fungal host specificity and simulating the effects of N deposition on endophytic fungal communities will help us better identify the ecological and evolutionary constraints that limit symbiont distribution. To fill this knowledge gap, we established the following three hypotheses: (1) In alpine meadows on the QTP, FEFs vary substantially among different host plant species, (2) a subset of microbial taxa will be shared among all host plant species, and (3) FEF communities vary substantially among plots with experimentally elevated levels of N under N-deficient conditions, and nutrient availability would have a greater influence in structuring fungal communities than host identity. We assume that if phylogenetically conserved traits serve as biological filters for endophyte colonization and establishment, the endophytic fungal community may vary considerably in species with different morphology, structure, and tissue texture, as suggested by Zhang and Yao (2015). A study in southern Chile, on the other hand, found that the homogeneity of endophytic fungal communities might be due to the filtering effect of physical and chemical traits of leaves independent of the evolutionary history of the host (Gonzalez-Teuber et al., 2020). Therefore, we hypothesize that in alpine meadows on the QTP, where productivity is strongly limited by N availability (Xu et al., 2015), N will transcend host identity in shaping the community composition of FEFs. To address these hypotheses, we used high-throughput sequencing to characterize the FEF community composition of three grass species in 4-year N addition treatments in an alpine meadow ecosystem on the QTP. To our knowledge, this is the first study to explore the responses of FEF communities to N fertilization in an alpine meadow ecosystem. This research provides better understanding of the response of plant-associated fungi to changing global scenarios.

## Materials and methods

### Study site and sample collection

This study was conducted in 2014 at the Research Station of Alpine Meadow and Wetland Ecosystems of Lanzhou University, Maqu County, China. The study site is located in the eastern QTP (33°40′N, 101°51′E, altitude 3,500 m a.s.l, Supplementary Figure S1), with an average precipitation of 620 mm (mostly occurs in summer). The mean annual temperature is about 1.2°C (ranging from −10°C in winter to 11.7°C in summer), and the frost-free season lasts about 95 days (Wu et al., 2010). The mean aboveground primary productivity is 280–400 g m^−2^ (dry weight), and the species richness is, on average, 20–35 per 0.25 m^2^ (Yang et al., 2011). The vegetation of the meadow is mainly dominated by *Kobresia capillifolia* (Cyperaceae), *Elymus nutans* Griseb (Poaceae), and *Anemone rivularis* Buch.-Ham. (Ranunculaceae). The soil type in the study area is Mattic Cryic Cambisols (Gong and Li, 2001). Total N deposition in this region is estimated to range from 14.26 to 18.65 kg ha^−1^ yr^−1^ (Lu and Tian, 2007).

The experimental site was used for grazing in the past and was fenced in 2011 to prevent grazing by large animals, such as yak and sheep. To understand how plants and related microorganisms respond to N addition in the field, NH_4_NO_3_ fertilizer was applied annually at the beginning of the growing season (usually in May) since then. The field experiment was a complete randomized block design, with 24 plots (20 × 10 m each, 1-m buffer strips) arranged in a regular 6 × 4 matrix. N gradients were generated with 0, 5, 10, and 15 g N (NH_4_NO_3_) m^−2^ yr^−1^ referring to N0 (control), N5, N10, and N15 treatments, and each treatment had six replicates (Supplementary Figure S1). After 4 years of N addition, available soil N concentration was increased significantly (*F* = 11.1, *p* < 0.001, Supplementary Table S1).

Samples were collected on 25 July 2014. We targeted three commonly observed plant species, namely, *A. rivularis, E. nutans*, and *Thermopsis lanceolata*. In each plot, six individuals of each species were randomly selected. For individuals of *A. rivularis* and *T. lanceolata*, five random mature leaves devoid of visible pathogen damage were collected from each individual. For individuals of *E. nutans*, the second leaf from the bottom of each individual was collected. The leaves collected from the same species in a plot were mixed as a sample. In total, we collected 71 samples (3 species × 24 plots, *T. lanceolata* was not found in one of the N15 plots). The samples were placed in zip-lock plastic bags and stored in coolers equipped with ice packs, transferred to the laboratory in 18 h, and stored at 4°C.

### Sample preparation, DNA extraction, and Illumina sequencing

The leaves were thoroughly washed in tap water, patted dry, and divided into two subsamples: One subsample was sterilized for DNA extraction within 36 h of collection, and the other was stored at −20°C until the determination of the physical and chemical properties of the leaves. Before DNA extraction, the leaves were immersed in 75% ethanol for 1 min, in 1% sodium hypochlorite for 2 min, and in 75% ethanol for 30 s to eliminate microorganisms on leaf surfaces. The surface-sterilized tissues were then rinsed with sterile water for 30 s three times and then patted dry with a sterile filter paper. Before sample grinding, ~1 ml of the final rinse water of each sample was plated on potato dextrose agar (PDA) and cultured in the dark to validate the effect of surface sterilization. The leaves were ground with liquid nitrogen using mortars and pestles in a sterile room. The mortars and pestles were sterilized before use and re-sterilized between samples to avoid cross-contamination.

Total genomic DNA was extracted using 100 mg leaf tissue powder from each sample using a Plant DNA Extraction Kit following the manufacturer's instructions (Tiangen Biotech, Beijing, China). The extracted DNA samples were frozen and shipped to Majorbio Bio-Pharm Technology Co., Ltd., Shanghai, China. Fungal diversity was determined by sequencing the internal transcribed spacer (ITS) region with primers ITS1F (CTTGGTCATTTAGAGGAAGTAA)/ITS2 (GCTGCGTTCTTCATCGATGC) (Gardes and Bruns, 1993) on an Illumina MiSeq platform (2 × 250 PE).

### Sequence processing

Sequences were pre-processed using the Trimmomatic tool (v 0.36, Bolger et al., 2014). 3′ or 5′ ends with a Phred quality score lower than 20 were trimmed, and sequences of <200 bp in length and with an average quality score of <20 on a window of 50 bases were discarded. The remaining sequences were imported into QIIME2 version 2019.10 for bioinformatics analyses (Bolyen et al., 2019). The qiime2-dada2 plugin was used for denoising, dereplication, merging paired-end reads, and removing chimeras (Callahan et al., 2016). Molecular singletons were removed from the downstream analysis to minimize the possibility of sequencing artifacts (Unterseher et al., 2011). Taxonomic assignments were determined using the qiime2-feature-classifier (Bokulich et al., 2018) classify-sklearn against species hypotheses (SH) of UNITE's database version 8.2 (Koljalg et al., 2020). Click or tap here to enter text. Trained with the Naive Bayes classifier with a confidence threshold of 97%. All representative sequencing data were submitted to the National Center for Biotechnology Information (NCBI) under accession numbers OM744714-OM745695.

### Leaf trait collection

The leaf traits were measured using the second subsample stored at −20°C. This work was completed within 10 days of sample collection. We focused on nitrogen and carbon fractions. Total leaf carbon and nitrogen per tree were determined using a C/N elemental analyzer, Vario MAXCN (Elementar, Hessia, Germany). Low-molecular weight (LMW) carbohydrates (mostly glucose, fructose, and sucrose) and high-molecular weight (HMW) carbohydrates (mainly fructans) were extracted and quantified from aboveground tissues, as previously described (Hunt et al., 2005; Rasmussen et al., 2007). Total free amino acids were analyzed from the ethanol fraction used for carbohydrate extraction and were determined colorimetrically with ninhydrin, as described previously (Yemm et al., 1955). Soluble proteins were extracted using 0.1% mercaptoethanol in 100 mM potassium phosphate buffer. Extracts were centrifuged, and the resulting supernatant was analyzed according to Bradford (1976) with absorbance measured at 595 nm using bovine serum albumin (BSA) as a standard.

### Statistical analyses

The averages and standard deviations of the tree traits and leaf chemicals in each plot were calculated. Diversity assessment used Fisher's alpha (a richness index) and Shannon-index (considering both richness and abundance). Analyses were combined with ANOVA of the multivariate generalized linear models and randomized species accumulation curves. The abundance-based Bray–Curtis similarity coefficient was used to examine the dissimilarity of community composition and turnover. The distinctiveness of leaf endophytic fungi in different hosts and under different N conditions, and independence of the interactions of hosts and N were tested using a permutational multivariate analysis of variance (PERMANOVA) using the distance metrics mentioned earlier. The beta diversity of FEFs was analyzed using non-metric multidimensional scaling (NMDS) by R 4.1.1 statistical software (R Core Team, 2019). In addition, the environmental variables were screened using bioenv, mantel, and *vif.cca* functions, and the selected environmental variables were fitted onto the NMDS ordination plot using the *envfit* function in the vegan package v2.5 (Oksanen et al., 2019). A Mantel test was used in the vegan package to identify the plant traits that significantly correlated with the FEF community composition. Pearson's correlation analyses were used to identify the plant traits that significantly correlated with the FEF diversity indices in R. To reflect the OTU composition similarity among different samples or host identities, we performed network analysis. Sample-level OTUs by occurrence frequency were first filtered. A correlation between the two samples was considered statistically robust if Spearman's correlation coefficient (ρ) was >0.4 and the *P*-value was <0.05. All the robust correlations identified from pairwise comparison of OTU abundance form a correlation network, where each node represents one sample and each edge stands for a strong and significant correlation between nodes. Network analyses used the R packages vegan, igraph (Csardi and Nepusz, 2006), and Hmisc (Harrell and Dupont, 2018). Network visualization was then conducted using the interactive platform Gephi (Bastian et al., 2009). All variables were transformed, where necessary. Prior to all analyses, data were rarefied to 1,944 sequence counts (minimum sequencing depth) using *rrarefy* function in the vegan package.

## Results

### Fungal OTU summary statistics

In total, 71 genomic DNA samples were sequenced, generating a total of 1,284,298 sequences after quality filtering. A total of 1,457 OTUs were clustered at the 97% sequence identity level. Of these, 475 (32.6%) OTUs were non-fungal taxa and were removed from the pool, the remaining 966,780 sequences; varied between 1,944 and 49,914 per sample; and clustered into 982 OTUs (Supplementary Figure S2a). Specifically, 337,614 sequences were observed in *A. rivularis*, 509,880 sequences were found in *E. nutans*, and 119,286 sequences were recovered in *T. lanceolata*, clustered into 505, 507, and 280 OTUs, respectively (Supplementary Table S2). The rarefaction curves of the observed OTU richness failed to approach the asymptote (Supplementary Figure S2b). This implies that a few rare fungal taxa were not observed, due to either insufficient sampling or sequencing depths. Nevertheless, valid comparisons can still be drawn at the species or at the plot level.

**Figure 1:**
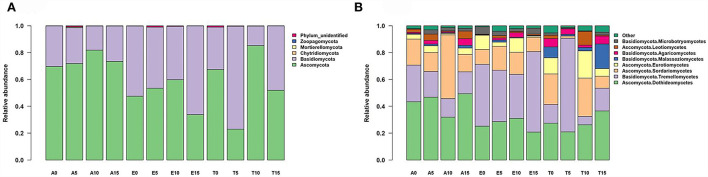
Taxonomic composition, **(A)** phylum level, and **(B)** class level of the fungal communities recovered from leaf interiors of three species along the nitrogen enrichment gradients in the study site. Bars show relative abundance of different taxonomic groups. The fungal endophyte classes with relative abundances of <0.1% were assigned to “Other”. A, *Anemone rivularis*; E, *Elymus nutans*; T, *Thermopsis lanceolata*. 0 (control), 5, 10, and 15 referring to 0, 5, 10, and 15 g N (NH_4_NO_3_) m^−2^ yr^−1^ treatments, respectively.

Endophytic fungal communities were strongly dominated by ascomycetes and basidiomycetes, together accounting for 99.4% of the sequences (Figure 1A; Supplementary Figure S3a; Supplementary Table S3). Members of those classified as “Basidiomycota” were present primarily in *E. nutans* and *T. lanceolata*, but ascomycetes are more prevalent in *A. rivularis* (Figure 1A). Early diverging lineages, including Chytridiomycota, Mortierellomycota, and Zoopagomycota (862 sequences, comprising 0.09% of the total sequences), were also observed (Supplementary Table S3). Unidentified OTUs (i.e., those classified as fungi but failed to be assigned to a phylum) comprised 0.51% of the total sequences (Supplementary Table S3). The most dominant class across our dataset, Dothideomycetes, comprised ~35% of all assigned sequence reads, followed by Tremellomycetes (29.2%), Sordariomycetes (16.0%), and Eurotiomycetes [(6.1%), Figure 1B; Supplementary Figure S3b; Supplementary Table S4].

### Fungal diversity patterns and community composition

**Figure 2:**
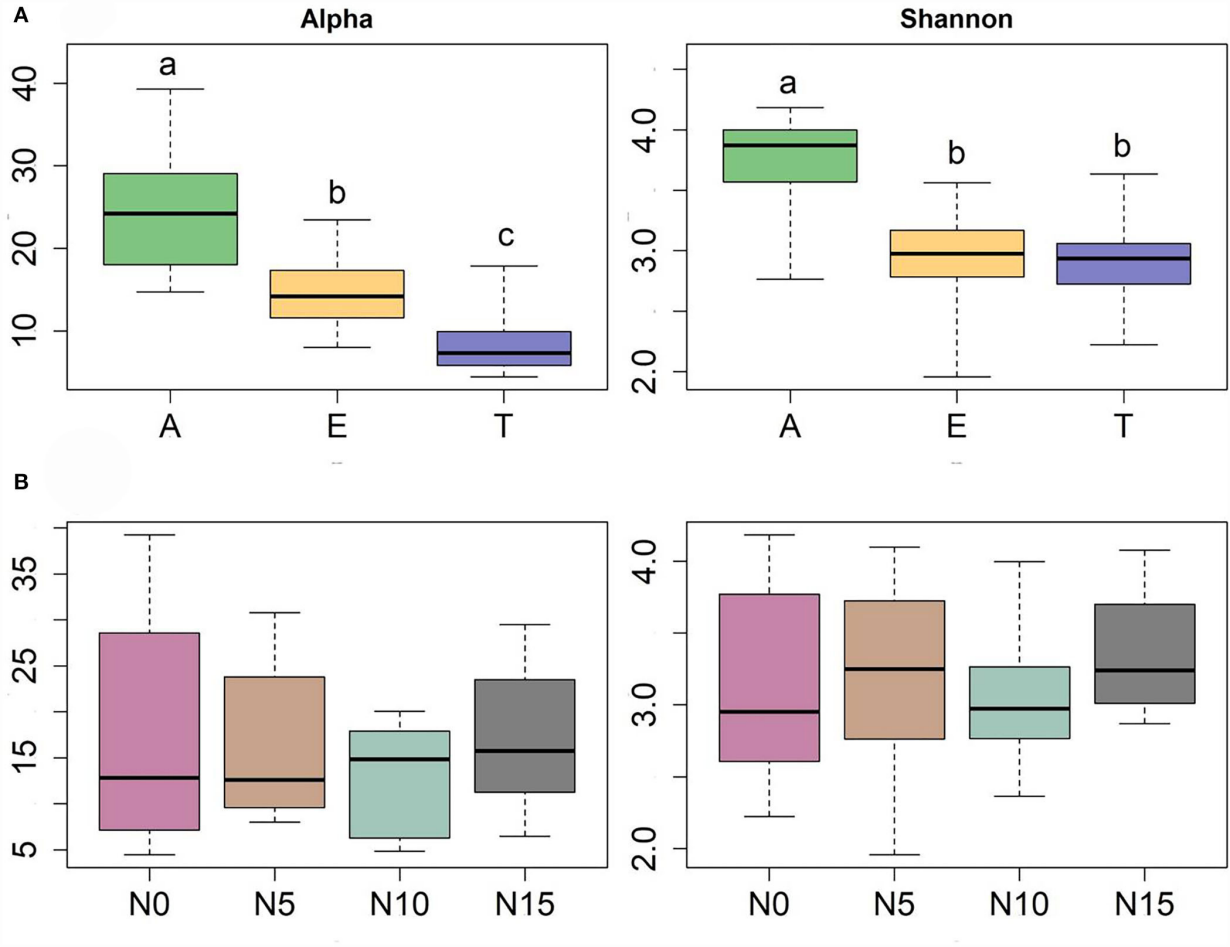
Diversity patterns of the entire fungal community of three species against host **(A)** and N gradients **(B)** based on linear models. Alpha is the number of observed species, and Shannon is the Shannon diversity index. The sequences were rarified to 1,944, *n* = 71. Significant differences of each variable are indicated by dissimilar letters above boxes. A, *Anemone rivularis*; E, *Elymus nutans*; T, *Thermopsis lanceolata*. N0, N5, N10, and N15 referring to 0, 5, 10, and 15 g N (NH_4_NO_3_) m^−2^ yr^−1^ treatments, respectively.

**Figure 3:**
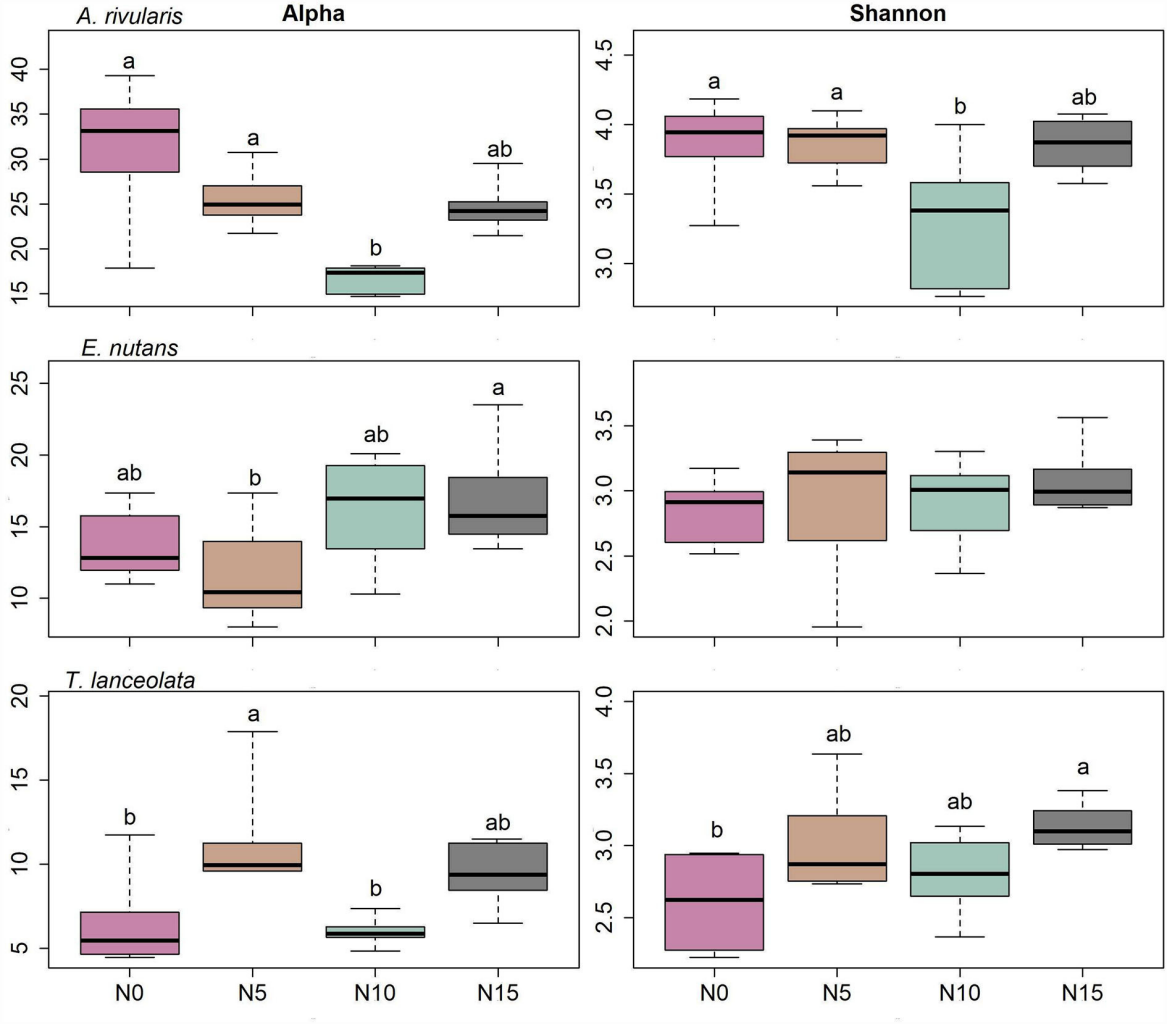
Diversity patterns of the fungal community within each host against N gradients. Alpha is the number of observed species, and Shannon is the Shannon diversity index. The sequences were rarified to 1,944, *n* = 71. Significant differences of each variable are indicated by dissimilar letters above boxes. N0, N5, N10, and N15 referring to 0, 5, 10, and 15 g N (NH_4_NO_3_) m^−2^ yr^−1^ treatments, respectively.

Diversity measures displayed a significant difference among the three hosts. *A. rivularis* samples showed higher phylotype richness and Shannon diversity than *E. nutans* and *T. lanceolata* (Figure 2A). Statistical tests confirmed a significant effect of host types (GLM, *p* < 0.01, Table 1). N input showed a relatively weak effect on fungal diversity (Figure 2B; Table 1). However, at a smaller scale (i.e., within-host scale), N input decreased the fungal diversity of *A. rivularis* but increased that of *T. lanceolata* (Figure 3; Supplementary Table S5). Thus, fungal diversity can be maintained across the plot scale, except at the smaller, within-host scale under the background of N enrichment.

**Table 1:** Diversity statistics for the fungal endophytes in three hosts based on a multispecies generalized linear model.

	**DF**	**Fisher's alpha**			**Shannon**		
		**Sum of squares**	**Dev**	***p***	**Sum of squares**	**Dev**	***p***
**Entire community**							
Host	2	3,059.3	196.1	**0.001**	10.9	3.3	**0.001**
N input	3	190.4	12.7	**0.012**	1.2	0.4	0.399
Host × N input	6	562.7	30.9	**0.001**	1.2	0.4	0.864

Significance at 0.05 is highlighted in bold.

**Figure 4:**
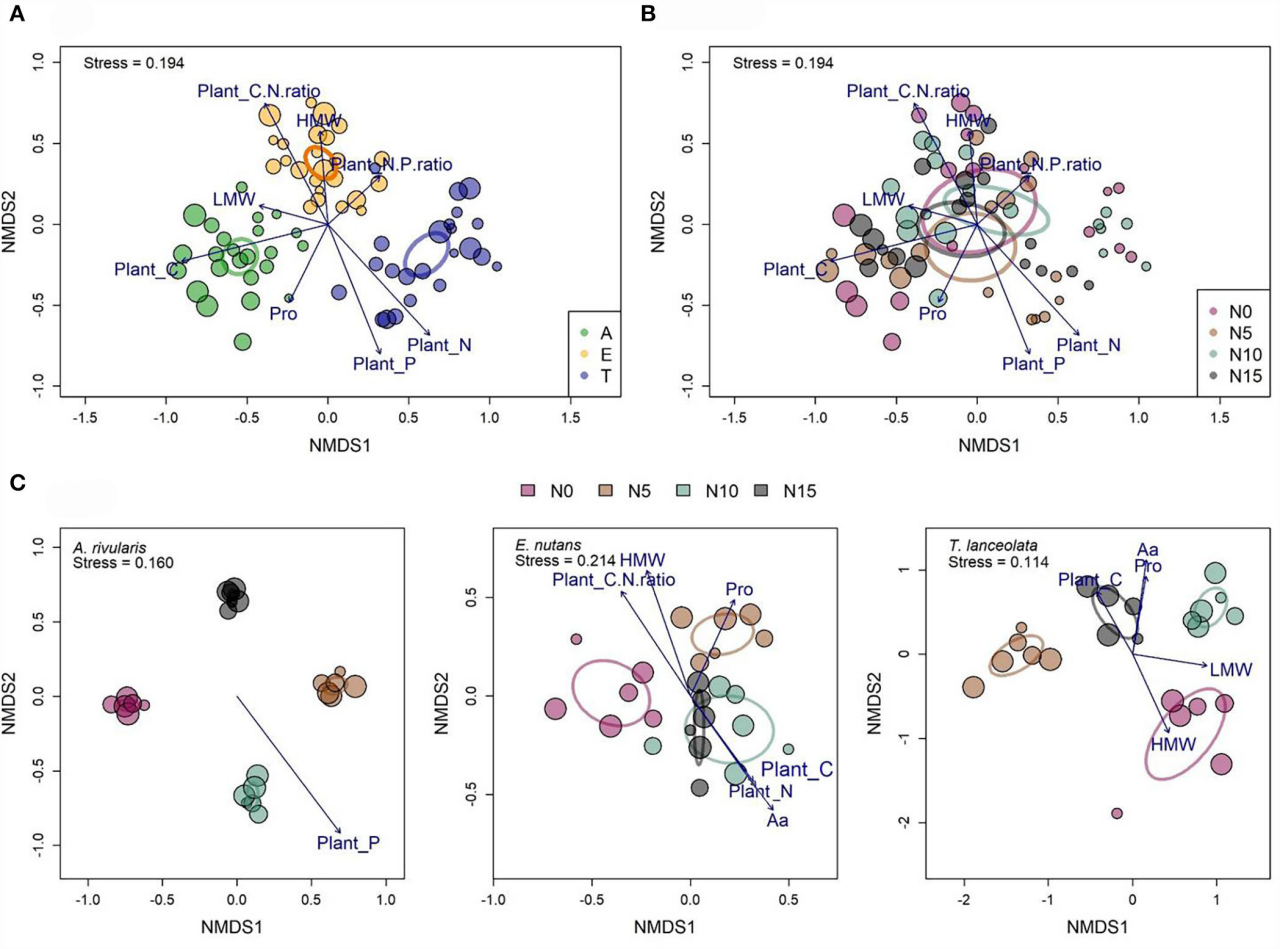
Community structure of fungal endophytes among different hosts **(A)**, N gradients **(B)**, and within each host along the N gradients **(C)** were assessed with non-metric multidimensional scaling (NMDS) (Bray–Curtis distance). Dot size is proportional to the operational taxonomic unit (OTU) richness of individual samples (*n* = 71). Ellipses with different colors indicate 95% confidence intervals. Significant plant variables that are correlated with each community ordination are shown. Pro: soluble proteins. LMW, low-molecular weight carbohydrates; HMW, high-molecular weight carbohydrates; Aa, total free amino acids; A, *Anemone rivularis*; E, *Elymus nutans*; T, *Thermopsis lanceolata*. N0, N5, N10, and N15 referring to 0, 5, 10, and 15 g N (NH_4_NO_3_) m^−2^ yr^−1^ treatments, respectively.

Composition of leaf mycobiome differed significantly among hosts (Figure 4A). However, the effects of fertilization led to overlapped fungal communities across the plot scale (Figure 4B). The dissimilarities of communities of FEFs among hosts, N gradients, and the interaction of the two factors were also confirmed by a multispecies generalized linear model calculation and a permutational multivariate analysis of variance (PERMANOVA) (Table 2). Community turnover in each host showed stronger clustering with N enrichment (Figure 4C; Supplementary Table S6).

**Table 2:** Statistical testing of fungal compositional dissimilarity based on a multispecies generalized linear model calculation (multispec.glm) and a permutational multivariate analysis of variance (PERMANOVA) using Bray–Curtis distance metrics.

	**Multispec.glm**		**Permanova**		
	**Dev**	***p***	***F***	***R* ^2^**	***p***
**Entire community**					
Host	6,053	**0.003**	21.936	0.248	**0.001**
N input	5,036	**0.003**	7.959	0.136	**0.001**
Host × N input	3,259	**0.003**	8.353	0.283	**0.001**

Significance at 0.05 is highlighted in bold.

### Plant functional trait variance along the N gradient

The effects of fertilization on plant measures varied among hosts. Only total free amino acids (Aa) and total soluble proteins (Pro) significantly changed in all three species with N fertilization (Supplementary Table S7). In *E. nutans*, both low-molecular weight (LMW) carbohydrates and high-molecular weight (HMW) carbohydrates decreased under high N treatment (*F* = 3.783, *p* = 0.027 and *F* = 3.253, *p* = 0.043, respectively). Although these water-soluble carbohydrates also decreased in *A. rivularis* and *T. lanceolata* under high N conditions, only HMW sugars in *T. lanceolata* showed a significant response (*F* = 7.547, *p* < 0.001).

Unlike observations in soil (Supplementary Table S1), only tissue N in *E. nutans* was found increased under the high N condition (*F* = 14.298, *p* < 0.001), leading to a significant decrease in the tissue C/N ratio (*F* = 18.287, *p* < 0.001) and an increase in the tissue N/P ratio (*F* = 4.129, *p* = 0.02). An increased tissue N/P ratio was observed in *A. rivularis* (*F* = 5.054, *p* = 0.009) due to a decreased tissue P concentration (*F* = 4.837, *p* = 0.011). At the plot scale, elevated nutrient supply can cause a significant change in Pro (*F* = 3.242, *p* = 0.04), Aa (*F* = 51.364, *p* < 0.001), and tissue N/P ratio (*F* = 5.854, *p* = 0.005).

### Effect of environmental variables on fungal diversity and community composition

Clearly, environmental variables were significantly correlated with both endophytic fungal diversity and community composition (Table 3). Pearson's correlation analyses showed that Pro, LMW sugars, tissue C, N, C/N ratio, and N/P ratio could be significantly related to the fungal alpha diversity indices to varying degrees (Table 3). There was a strong positive correlation between leaf carbon and endophytic fungal diversity indices, and LMW carbohydrates and Pro also showed a similar effect. Tissue N had a significant negative influence on endophytic fungal richness, but not on Shannon diversity. In addition, the leaf N/P ratio is negatively related to fungal diversity.

**Table 3:** Pearson's product–moment correlations between environmental variables and alpha (Fisher and Shannon indices) and beta diversity (Bray–Curtis distances) of fungal endophytes in three hosts.

**Pearson *r***	**Fisher's alpha**	**Shannon**	**Beta diversity**
Pro	0.388**	0.471***	0.060
LMW	0.315**	0.285*	0.460
HMW	−0.112	−0.214	−0.010
Aa	−0.083	0.001	0.039
Plant_N	−0.466***	−0.188	0.373***
Plant_C	0.685***	0.570***	0.416***
Plant_P	−0.184	0.074	0.261***
Plant_C.N.ratio	0.251*	0.022	0.233***
Plant_N.P.ratio	−0.343**	−0.368**	0.006

The tested environmental variables included Soluble Pro, proteins; LMW, low-molecular weight carbohydrates; HMW, high-molecular weight carbohydrates; Aa, total free amino acids; plant total nitrogen; C, carbon; P, phosphorus; C/N ratio; and N/P ratio. Pearson's correlation coefficients (r) are shown.

* P < 0.05.

** P < 0.01.

*** P < 0.001.

The Mantel test revealed a significant correlation between the fungal community composition and the following environmental variables (listed from highest to lowest Pearson's correlation coefficients): tissue C > N > P > C/N ratio (Table 3). With the *envfit* function in the vegan package (Oksanen et al., 2019) of R, Pro, LMW sugars, tissue C, N, P, C/N ratio, and N/P ratio were chosen to fit the NMDS plot (Figures 4A,B). When controlling for species, OTU assemblage was significantly correlated with tissue P in *A. rivularis*. Pro, Aa, HMW, tissue C, N, and C/N ratio were correlated with the fungal community in *E. nutans*, and ordination of *T. lanceolata* displayed the significance of five parameters, namely, Aa, Pro, LMW, and HMW sugars, and tissue C (Figure 4C). In summary, among all the measured indices, leaf carbon was considered the most significant factor constraining alpha and beta diversity.

### Core microbiome and fungal co-occurrence network

Venn diagrams illustrated 927 OTUs (rarefied data) partitioning among the plant species and N gradients (Supplementary Figure S4). A total of 94 OTUs were shared among three species (10.14%, Supplementary Figure S4a), and 95 OTUs appeared in all treatment plots (10.25%, Supplementary Figure S4b). At the within-host scale, 47 (9.46%), 58 (11.79%), and 18 (6.43%) OTUs in *A. rivularis, E. nutans*, and *T. lanceolata* were shared among all N gradients, respectively (Supplementary Figure S5). We found evidence for a core microbiome, whereby nine OTUs were present among 90% or more of samples in the N0 (control) plot. These nine OTUs comprised ~30% of the total reads (Supplementary Table S8), and taxonomic assignments were made using the National Center for Biotechnology Information (NCBI) nucleotide database. Most of the putative dominant fungi were previously found in close association with plants, which corroborated their potential symbiotic capability. In addition, among the 10 OTUs, seven were found among 90% or more of all individuals in all plots, indicating their prevalence in varied hosts along the environmental gradient.

**Figure 5:**
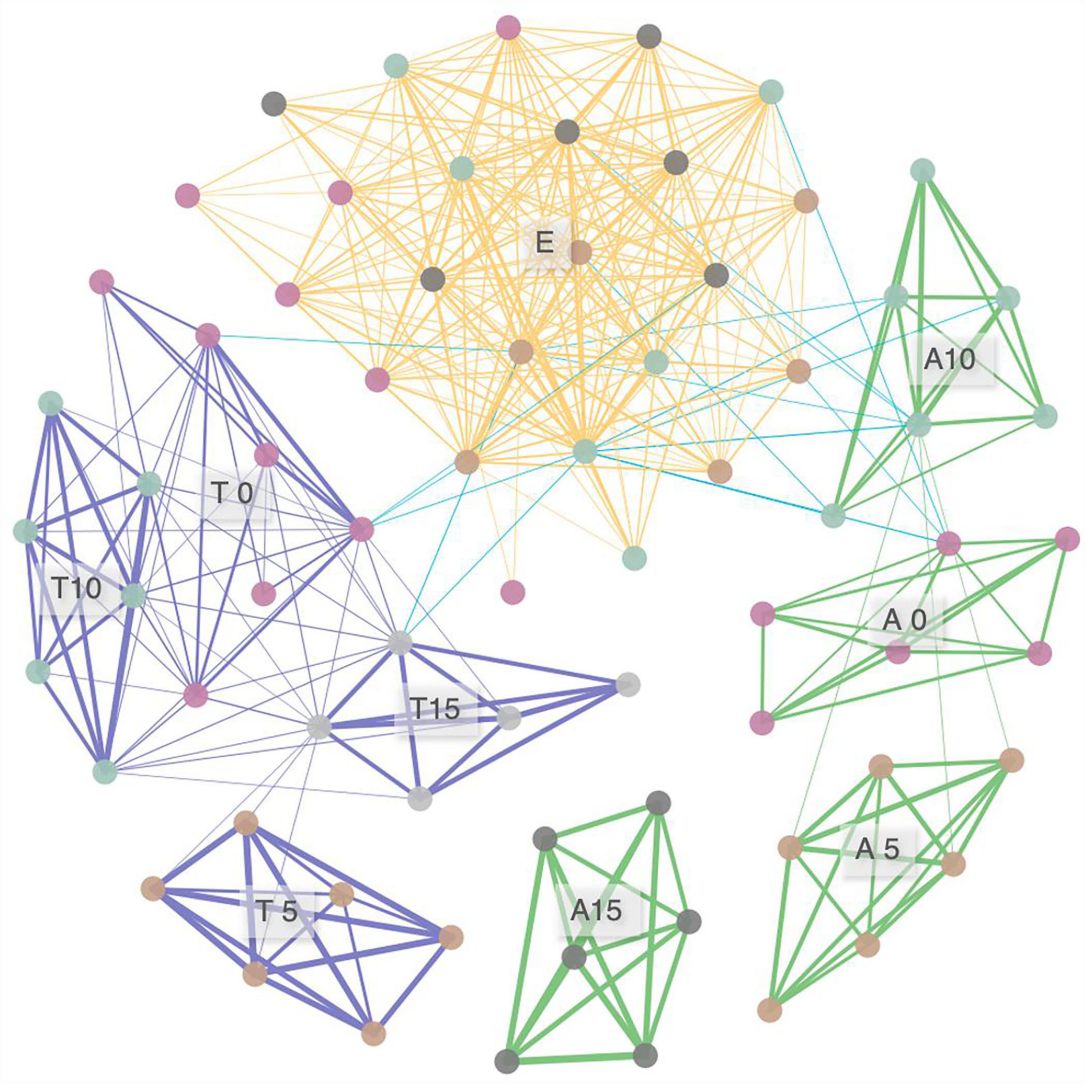
Networks of taxa similarity in three species along the nitrogen enrichment gradients, based on correlation analysis. A connection stands for a correlation with Spearman's ρ > 0.4 and *p* < 0.05. The thickness of each connection between two nodes (i.e., edge) is proportional to the value of Spearman's correlation coefficients ranging from 0.40 to 0.93. A, *Anemone rivularis*; E, *Elymus nutans*; T, *Thermopsis lanceolata*. 0 (control), 5, 10, and 15 referring to 0, 5, 10, and 15 g N (NH_4_NO_3_) m^−2^ yr^−1^ treatments, respectively.

For the network analysis of FEFs, 378 pairs of significant correlations were identified from 71 samples (Figure 5). Nodes of *A. rivularis* and *T. lanceolata* were highly interconnected (clustered) at each N level. Unique fungal taxon co-occurrence patterns were also found in *E. nutans* but were not affected by N input. Inter-host co-occurrence patterns were also observed; however, the correlations were relatively loose (18 edges), with Spearman's correlation coefficients ranging from 0.40 to 0.44. Overall, co-occurring FEFs tend to be tightly related to host identity, and the effect of N varied among species.

## Discussion

### Communities of FEFs in alpine meadows are highly diverse

*E. nutans* is dominant perennial grass in the QTP due to its high adaptability (Miao J. et al., 2011; Miao Q. et al., 2011), and *A. rivularis* and *T. lanceolata* are widespread forbs in this area. A recent survey on 596 publications on endophyte biodiversity revealed that forbs and graminoid-inhabiting fungal endophytes are largely understudied, especially in multiple hosts (Harrison and Griffin, 2020). In this study, diverse FEFs were found in three herbaceous hosts, suggesting a complex fungal network within QTP plant tissues, despite the geographic isolation and extreme environmental conditions. In a recent study, 210 endophytic fungal OTUs were identified in *E. nutans* collected from a nearby field site using similar study methods (Guo et al., 2021). Here, we identified 576 OTUs (492 OTUs after data rarefaction) in leaves of *E. nutans*, challenging the argument put forward by Harrison and Griffin (2020) that roots were the tissue that harbors the richest endophytes in graminoids. In terrestrial plants, environmental differences were proven to contribute to differences between organs in endophyte flora (Fisher et al., 1992). Higher fungal richness in leaves of *E. nutans* might be caused by the more dynamic aboveground environment, since environmental variability could promote diversity (Hutchinson, 1961; Chesson, 2000). During the short growing season in the QTP, leaves are more biochemically active than roots and may provide more available nutrients to attract microorganisms. Alternatively, low temperature can limit microbial activity, reducing the transmission of endophytes in QTP soil. Given the limited amount of published data on endophytes of alpine plants, it is difficult identify which patterns of distribution are normal.

To our knowledge, this is the first study to apply high-throughput sequencing to explore the endophyte communities in *A. rivularis* and *T. lanceolata*. Here, a taxonomically diverse assemblage of FEFs was recovered from *A. rivularis*, whereas by cultivation-dependent methods, only 23 fungal taxa were isolated from the leaves of Anemone tomentosa in a study in west China. We screened relatively less fungal taxa in *T. lanceolata* (280 OTUs), possibly due to the reduced abundance of *T. lanceolata* after N enrichment in the study site (Jiang et al., 2018). Abundant hosts have been proven to support a greater number and diversity of symbiotic fungi (Gilbert et al., 2007). Thus, we suggest that host abundance plays an important role in structuring endophytic microbiomes.

Ascomycota was described as the predominant endophytic fungal phyla in biomes ranging from tropical rainforests to harsh Antarctica in previous works (Arnold and Lutzoni, 2007; Zhang and Yao, 2015; Teasdale et al., 2018; Whitaker et al., 2020). Using 454 pyrosequencing, only 0.4% endophytic Basidiomycota was recovered from leaves of *Metrosideros polymorpha* in the tropics (Zimmerman and Vitousek, 2012). Conversely, 8.6% endophytic Basidiomycota was found in subalpine timberline ecotone (Yang et al., 2016), and 19.4% of FEFs in four species in a high arctic zone are basidiomycetous fungi (Zhang and Yao, 2015). Research on fungal endophytes has expanded dramatically in recent years, but the ecological roles of endophytic basidiomycetes are still unknown. Here, we found as high as 36.75% endophytic Basidiomycota, adding evidence to the importance of Basidiomycota in high-altitude or high-latitude areas.

### Effects of N modifications at plot and within-host scale

The impact of human development on endophyte biodiversity in wilderness areas remains understudied (Harrison and Griffin, 2020). We hypothesized that N could lead to significant community turnover at both plot and within-host scale. We found that fungal diversity for all samples (or all individuals) pooled was not dominantly shaped by N supply at the plot scale after 4 years of fertilization treatment (although some indices showed a significant effect of N, clearly lower deviances, *F*, and *R*^2^ statistics were observed, cf. Tables 1, 2; Figures 2, 4). The findings agree with culture-based experimental evidence from Sweden recently (Witzell et al., 2022) that N input did not alter the total richness and Shannon diversity of FEFs in 12 aspen genotypes. We suggest that in the alpine meadow ecosystems, total fungal communities harbored in diverse groups of plants tend to persist under increasing N deposition. However, it is still not clear if plant microbiomes could be affected by anthropogenic environmental changes at larger spatial scales across the QTP landscapes. Further studies incorporating additional sites (or testing for spatial autocorrelation) are required in the QTP in order to make a strong inference or generalizations on these fungal communities.

In the present study, the effects of elevated N are more pronounced at the within-host scale (Supplementary Tables S5, S6; Figures 3–5). Fungal assemblages of *A. rivularis* exhibited decreased species diversity (Shannon diversity and richness) across the N enrichment gradients. However, an increased fungal diversity pattern was observed in *T. lanceolata* as compared to the control group. By contrast, FEF communities of *E. nutans* were not significantly affected by N. A study of root-associated arbuscular mycorrhizal (AM) fungi in *E. nutans* within the same experimental plots sampled at the same time found that N addition reduced AM fungal abundance (Jiang et al., 2018). These findings indicate that the effects of N input on plant microbiomes are multifarious and may depend upon host identity and tissue types. The multispecies generalized linear model, PERMANOVA model together with NMDS, and network analyses demonstrated that endophyte community composition varies significantly among host species, supporting findings of previous studies (e.g., Arnold and Lutzoni, 2007; Higgins et al., 2007; Dastogeer et al., 2018; Liu et al., 2019; Chen et al., 2020). Perennial hosts were suggested to cultivate and nurture their microbial surroundings (Baltrus, 2017). Thus, it was possible that in the stressful environment in the QTP, the fungal communities may have adapted to different types of leaves over evolutionary timescales. We reinforce the view that host plant identity is the significant driver of endophyte assemblage patterns, and we suggest host identity supersedes N supply in shaping the community composition of FEFs.

### Host tissue C and related compounds significantly correlated with FEF diversity

Consistent with previous studies (Rasmussen et al., 2007; Ryan et al., 2014), leaf carbohydrates (LMW and HMW sugars) decreased with N input. Leaves provide shelter and nutrition for endophytes, and foliar carbon was known to correlate with plant function (Wright et al., 2004) and life history strategy (Wright et al., 2010), yet how leaf chemicals, especially leaf carbon, affect communities of fungal endophytes is not well studied. We found a strong relation between tissue carbon and communities of FEFs in the study site (Table 3). The NMDS plot with fitted environmental variables also showed that the carbon-associated factors, including tissue C, C/N ratio, and LMW and HMW sugar, significantly correlated with FEF community assemblages along the N gradient (Figures 4A,B). In addition, these carbon-associated factors correlated well with the dissimilarities among fungal assemblages in *E. nutans* and *T. lanceolata*. Similar trends were also found in the leaves of *Betula* (Yang et al., 2016) and *Ficus* (Liu et al., 2019), emphasizing the universal relationship between carbon source availability and this heterogeneous ecological guild in plants.

### Core microbiomes exist among hosts and the N gradient

We demonstrated the existence of a fairly dominant core microbiome: three focal species shared one-third of all endophyte sequences, seven OTUs were prevalent in all sampled hosts along the N gradient, and two prevalent OTUs assigned to uncultured fungus in this study were also observed in the phyllosphere of trees in different ecosystems (Supplementary Table S8), indicating their potential wide niche range. Some subsets of fungi that comprise the core microbiome may have functional roles that enhance their ability to colonize the interior of a phylogenetically diverse group of plant species (Vandenkoornhuyse et al., 2015). These traits likely include high habitat adaptation (Pereira et al., 2019) and metabolite (enzymes or hormone) production that induces stomatal opening for fungi entry (Hardoim et al., 2015). The same microbial taxa may reach varied plant species in varied environmental conditions, and hence, searching for endophytes from the core microbiome of wild plants adapted to unhospitable habitats will provide solutions for the study of stress tolerance of plants.

## Conclusion

This study provides insights into the changes in plant fungal microbiome in response to host identity and environmental conditions. We report two key findings: first, in the harsh habitat of the Qinghai–Tibetan Plateau, host identity, rather than N supply, is a stronger factor for driving communities of foliar fungal endophytes, even in an alpine meadow ecosystem where productivity is strongly limited by N availability; and second, N addition has varying effects on both α and β diversity of within-host scale fungal endophytes. We demonstrated leaf carbon and carbohydrates significantly affected fungal communities in host leaves, indicating FEFs may play a pivotal role in the carbon cycling of alpine meadow ecosystems. In addition, we found unequivocal evidence for a core microbiome present among 90% or more of all control samples, representing one-third of the total reads. The results suggest that even though soil resource availability often mediates plant performance, environmental modifications on fungal microbiomes likely depend on the host species. We suggest that future studies quantify the nutritional requirements of endophytes, at both small and large scales. The results implement theoretical frameworks of community ecology and gain new sights into the dynamics of endophyte communities in grasses and forbs driven by environment and host functional traits.

## Data availability statement

The datasets presented in this study can be found in online repositories. The names of the repository/repositories and accession number(s) can be found below: https://www.ncbi.nlm.nih.gov/, OM744714:OM745695.

## Author contributions

Conceptualization: HF, QZ, and YL. Methodology: GD, HF, and YL. Software, formal analysis, and visualization: YM. Investigation: GS, YL, QZ, and YM. Resources: GD. Data curation: QZ and YM. Original draft preparation: HF, QZ, and YM. Project administration: QZ and HF. All authors contributed to manuscript revision, read, and approved the submitted version.

## Funding

This work was financially supported by the Second Tibetan Plateau Scientific Expedition and Research Program (2019QZKK0301) and the National Natural Science Foundation of China (31971445, 31870494, 31860146, 32171579, and U21A20186).

## Conflict of interest

The authors declare that the research was conducted in the absence of any commercial or financial relationships that could be construed as a potential conflict of interest.

## Publisher's note

All claims expressed in this article are solely those of the authors and do not necessarily represent those of their affiliated organizations, or those of the publisher, the editors and the reviewers. Any product that may be evaluated in this article, or claim that may be made by its manufacturer, is not guaranteed or endorsed by the publisher.
