# Supplementary material for: Can nitrogen supersede host identity in shaping the community composition of foliar endophytic fungi in an alpine meadow ecosystem?
Source: Front Microbiol. 2022 Aug 22;13:895533. doi: 10.3389/fmicb.2022.895533 (PMC9441931; doi:10.3389/fmicb.2022.895533)
Supplement: Supplementary file 1 [file Data_Sheet_1.docx]

Table S1 Soil characteristics (mean ± SE) in the N gradients in the study site. N0, N5, N10 and N15 represent fertilizer applications of 0, 5, 10, 15 g N (NH_4_NO_3_) m-2 yr-1, respectively. Significant N effects are highlighted in bold. *p < 0.05, **p < 0.01, ***p < 0.001

|  | Moisture (%) | pH | Total N (%) | Organic C (%) | Available N (mg kg^-1^) | Available P (mg kg^-1^) | Available N: P ratio |
| --- | --- | --- | --- | --- | --- | --- | --- |
| N0 | 24.3 ± 0.8 | 5.3 ± 0.01 | 0.3 ± 0.01 | 1.9 ± 0.02 | 46.4 ± 3.7 | 8.7 ± 1.3 | 5.8 ± 0.8 |
| N5 | 24.6 ± 0.7 | 5.3 ± 0.04 | 0.3 ± 0.02 | 2.0 ± 0.11 | 40.0 ± 3.6 | 10.1 ± 2.5 | 5.5 ± 1.4 |
| N10 | 24.4 ± 0.2 | 5.2 ± 0.01 | 0.3 ± 0.01 | 2.0 ± 0.05 | 59.1 ± 5.2 | 7.9 ± 0.9 | 8.4 ± 1.7 |
| N15 | 24.4 ± 0.5 | 5.2 ± 0.02 | 0.3 ± 0.01 | 2.1 ± 0.06 | 78.51 ± 8.4 | 7.8 ± 1.2 | 11.1 ± 1.6 |
| Summary of N effect |  |  |  |  |  |  |  |
| F | 0.081 | **6.843** ** | 1.014 | 0.701 | **11.144** *** | 0.273 | **4.832** ** |

Table S2 Number of fungal sequences and Operational taxonomic units (OTUs) recovered in the three species in the study site. Rarefied data were generated with Rarefy function in the GUniFrac R package (Chen et al 2012) to 1,944 sequences to minimize the difference of sequencing depth across samples.

|  | Non-rarefied | |  | Rarefied | |
| --- | --- | --- | --- | --- | --- |
|  | Sequences | OTUs |  | Sequences | OTUs |
| Anemone rivularis | 337,614 | 505 |  | 46,656 | 497 |
| Elymus nutans | 509,880 | 567 |  | 46,656 | 492 |
| Thermopsis lanceolata | 119,286 | 280 |  | 44,712 | 280 |
| Total | 966,780 | 982 |  | 138,024 | 927 |

Table S3 Summary of fungal endophyte phyla of three species along a nitrogen enrichment gradient in the study site

|  | Sequences | Percentage | OTUs |
| --- | --- | --- | --- |
| Fungi | 996,780 | 100 | 982 |
| Dikaryon | 961,015 | 99.40 | 932 |
| Ascomycota | 605,688 | 62.65 | 651 |
| Basidiomycota | 355,327 | 36.75 | 281 |
| Non-Dikaryon | 862 | 0.09 | 14 |
| Chytridiomycota  Mortierellomycota | 16  781 | <0.01  0.08 | 1  12 |
| Zoopagomycota | 65 | <0.01 | 1 |
| Unidentified | 4903 | 0.51 | 36 |
| Total | 996,780 | 100 | 982 |

Summary includes the number of sequences, the percentage of the total number of sequences that each classification represented, and the number (based on 97% rDNA ITS1 similarity) of OTUs attributed to each taxon. The minimum confidence to record an assignment in RDP classifier was 0.8.

Table S4 Relative percent abundance and sequence counts of OTUs assigned to the most abundant classes (accounted for more than 1% of all sequences) across all samples

| Class | Sequence count | Relative abundance (%) |
| --- | --- | --- |
| Dothideomycetes | 351490 | 35.26 |
| Tremellomycetes | 291391 | 29.23 |
| Sordariomycetes | 159899 | 16.04 |
| Eurotiomycetes | 61143 | 6.13 |
| Leotiomycetes | 24678 | 2.48 |
| Microbotryomycetes | 21835 | 2.19 |
| Agaricomycetes | 15005 | 1.51 |
| Malasseziomycetes | 14766 | 1.48 |

Table S5 Diversity statistics for fungal community in each of the three hosts based on a multispecies generalized linear model. A: Anemone rivularis, E: Elymus nutans, T: Thermopsis lanceolata. Significance at 0.05 are highlighted in bold.

|  | DF | Fisher’s alpha | | |  | Shannon | | |  |
| --- | --- | --- | --- | --- | --- | --- | --- | --- | --- |
| N effect |  | Sum of suqares | Dev | p |  | Sum of suqares | Dev | p |  |
| A | 3 | 643.1 | 27.2 | **0.002** |  | 1.3 | 0.4 | 0.268 |  |
| E | 3 | 106.4 | 7.4 | 0.055 |  | 0.2 | 0.1 | 0.92 |  |
| T | 3 | 114.6 | 13.7 | **0.009** |  | 0.9 | 0.33 | 0.36 |  |

Table S6 Permutational multivariate analyses of variation of the compositional dissimilarity of fungal endophytes in each host along the N gradient. A: Anemone rivularis, E: Elymus nutans, T: Thermopsis lanceolata. N0, N5, N10 and N15 referring to 0, 5, 10 and 15 g N (NH_4_NO_3_) m-2 yr-1 treatments, respectively. Significance at 0.05 are highlighted in bold.

|  | Pairs | F | R^2^ | p |
| --- | --- | --- | --- | --- |
| A | N0 vs N5 | 12.227 | 0.550 | **0.002** |
|  | N0 vs N10 | 11.205 | 0.528 | **0.002** |
|  | N0 vs N15 | 16.266 | 0.619 | **0.007** |
|  | N5 vs N10 | 15.483 | 0.608 | **0.001** |
|  | N5 vs N15 | 25.216 | 0.716 | **0.002** |
|  | N10 vs N15 | 21.721 | 0.685 | **0.005** |
| E | N0 vs N5 | 2.541 | 0.203 | **0.003** |
|  | N0 vs N10 | 1.985 | 0.166 | **0.002** |
|  | N0 vs N15 | 2.369 | 0.192 | **0.002** |
|  | N5 vs N10 | 2.488 | 0.199 | **0.006** |
|  | N5 vs N15 | 2.969 | 0.229 | **0.004** |
|  | N10 vs N15 | 1.970 | 0.165 | **0.001** |
| T | N0 vs N5 | 12.316 | 0.552 | **0.003** |
|  | N0 vs N10 | 7.479 | 0.428 | **0.002** |
|  | N0 vs N15 | 8.227 | 0.478 | **0.005** |
|  | N5 vs N10 | 23.145 | 0.698 | **0.001** |
|  | N5 vs N15 | 21.161 | 0.702 | **0.001** |
|  | N10 vs N15 | 15.804 | 0.637 | **0.005** |

Table S7 Trait (mean ± SE) of different hosts along the N gradients in the study site. A: Anemone rivularis, E: Elymus nutans, T: Thermopsis lanceolata. N0, N5, N10 and N15 represent fertilizer applications of 0, 5, 10, 15 g N (NH_4_NO_3_) m-2 yr-1, respectively. Pro: Soluble proteins. LMW: Low molecular weight carbohydrates. HMW: high molecular weight carbohydrates. Aa: Total free amino acids. Values are the means ± SE. Significant N effects are highlighted in bold. *p < 0.05, **p < 0.01, ***p < 0.001.

| Host | Plot | Pro | LMW | HMW | Aa | Plant_N | Plant_C | Plant_P | Plant_C.N.ratio | Plant_N.P.ratio |
| --- | --- | --- | --- | --- | --- | --- | --- | --- | --- | --- |
| A | N0 | 13.10 ± 0.47 | 70.63 ± 1.52 | 6.21 ± 0.39 | 2.36 ± 0.08 | 17.55 ± 0.46 | 460.52 ± 0.70 | 2.12 ± 0.07 | 26.83 ± 0.76 | 8.58 ± 0.38 |
|  | N5 | 18.08 ± 1.34 | 58.92 ± 4.64 | 6.49 ± 0.61 | 2.86 ± 0.05 | 16.42 ± 0.49 | 462.86 ± 0.93 | 2.43 ± 0.05 | 28.86 ± 0.76 | 6.99 ± 0.39 |
|  | N10 | 10.45 ± 0.27 | 69.40 ± 2.97 | 8.69 ± 0.61 | 5.24 ± 0.16 | 18.93 ± 0.51 | 467.92 ± 1.18 | 2.22 ± 0.05 | 25.37 ± 0.81 | 8.71 ± 0.38 |
|  | N15 | 9.37 ± 0.65 | 56.52 ± 3.59 | 5.65 ± 0.50 | 8.67 ± 0.16 | 20.79 ± 0.63 | 465.14 ± 1.36 | 1.72 ± 0.05 | 22.90 ± 0.60 | 12.41 ± 0.50 |
| N effect | | |  |  |  |  |  |  |  |  |
| F |  | **3.992 *** | 0.756 | 1.030 | **93.668 ***** | 2.145 | 1.455 | **4.837 *** | 1.931 | **5.054 **** |
| E | N0 | 1.22 ± 0.15 | 56.16 ± 1.96 | 20.33 ± 2.18 | 2.41 ± 0.17 | 9.64 ± 0.22 | 441.97 ± 1.08 | 0.91 ± 0.02 | 46.48 ± 0.98 | 10.76 ± 0.37 |
|  | N5 | 10.58 ± 1.95 | 61.10 ± 3.33 | 17.58 ± 1.32 | 2.68 ± 0.09 | 9.57 ± 0.19 | 444.40 ± 0.97 | 1.01 ± 0.02 | 46.99 ± 0.95 | 9.57 ± 0.28 |
|  | N10 | 3.04 ± 0.29 | 49.79 ± 2.81 | 12.34 ± 1.39 | 5.46 ± 0.42 | 14.29 ± 0.43 | 449.56 ± 0.48 | 1.01 ± 0.03 | 32.37 ± 1.04 | 15.06 ± 1.00 |
|  | N15 | 2.14 ± 0.14 | 29.13 ± 3.46 | 5.39 ± 0.67 | 8.71 ± 0.17 | 17.32 ± 0.64 | 448.70 ± 1.19 | 1.11 ± 0.02 | 26.88 ± 0.90 | 15.59 ± 0.51 |
| N effect | | |  |  |  |  |  |  |  |  |
| F |  | **3.128 *** | **3.783 *** | **3.253 *** | **23.424 ***** | **14.298 ***** | 2.290 | 2.286 | **18.287 ***** | **4.129 *** |
| T | N0 | 6.19 ± 0.16 | 42.22 ± 2.52 | 5.89 ± 0.13 | 3.06 ± 0.17 | 35.48 ± 0.92 | 430.61 ± 0.88 | 2.73 ± 0.05 | 12.39 ± 0.32 | 13.27 ± 0.57 |
|  | N5 | 7.34 ± 0.25 | 27.53 ± 1.61 | 3.63 ± 0.13 | 4.50 ± 0.26 | 33.99 ± 0.65 | 437.09 ± 0.62 | 3.13 ± 0.05 | 13.00 ± 0.25 | 10.89 ± 0.21 |
|  | N10 | 9.62 ± 0.17 | 40.96 ± 1.39 | 3.79 ± 0.20 | 6.84 ± 0.32 | 34.88 ± 1.11 | 434.68 ± 1.09 | 3.03 ± 0.07 | 12.89 ± 0.46 | 11.65 ± 0.43 |
|  | N15 | 8.48 ± 0.33 | 30.85 ± 2.3 | 3.83 ± 0.20 | 6.95 ± 0.48 | 39.35 ± 1.13 | 438.71 ± 0.70 | 2.93 ± 0.08 | 11.36 ± 0.37 | 13.49 ± 0.33 |
| N effect | | |  |  |  |  |  |  |  |  |
| F |  | **7.690 **** | 2.391 | **7.547 **** | **6.787 **** | 0.964 | 2.842 | 1.419 | 0.694 | 1.565 |
| N effect on three hosts | | |  |  |  |  |  |  |  |  |
| F | | **3.242 *** | 2.224 | 1.975 | **51.364 ***** | 0.739 | 1.025 | 0.532 | 2.006 | **5.855 **** |

Table S8 BLAST-based taxonomic affinity of the nine Operational taxonomic units (OTUs) present in 90% or more of control samples (*n* = 18) of Anemone rivularis, Elymus nutans, and Thermopsis lanceolata. Items in bold are taxa found in 90% or more of all plant individuals (*n* = 71). Relative abundance is the percentage of total reads from control samples from a particular taxon. Coverage and similarity were derived from the results of the default megablast in NCBI Basic Local Alignment Search Tool.

| Relative Abundance | Best blast match to NCBI-nr database | | | | |
| --- | --- | --- | --- | --- | --- |
|  | GeneBank NO. | Coverage | % Identity | Definition | Ecological niche |
| 11.70 | FJ757520^1^ | 100 | 100 | uncultured fungus | *Quercus macrocarpa* phyllosphere fungi(Jumpponen & Jones 2009) |
| 5.76 | MK415876 | 100 | 100 | Fungal sp. | Grass foliar endophytes (Kivlin & Rudgers 2019) |
| 4.49 | JN906461 | 100 | 100 | uncultured fungus | *Fagus sylvatica* phyllosphere fungi (Cordier *et al.* 2012) |
| 1.63 | MW157700^2^ | 100 | 98.3 | *Hypoxylon* sp. | Asteraceae plants foliar endophytes (Whitaker *et al.* 2020) |
| 1.42 | MN054752 | 100 | 100 | *Cladosporium* sp. | *Populus trichocarpa* foliar endophytes (Barge 2019) |
| 1.24 | MZ066739 | 100 | 100 | *Alternaria alternata* | *Ageratina Adenophora* foliar endophytes (Wen et al., paper in submission) |
| 1.20 | MK190674^3^ | 100 | 100 | *Neoascochyta europaea* | Wheat foliar endophytes (Golzar *et al.* 2019) |
| 1.01 | JQ768938 | 93 | 100 | *Cryptococcus sp.* | Glacier surface snow (Tibetan Plateau) |
| 0.85 | MK336446 | 100 | 100 | *Malassezia restricta* | *Ipomoea pes-caprae* foliar endophyte (Yeh & Kirschner 2019) |

1 Can also be assigned to JN906065 uncultured fungus with more identity, which was a phyllosphere fungus with *Fagus* sylvatica ^(Cordier^ *^et al.^* ^2012)^.

2 Can also be assigned to GQ509809 uncultured fungus with more identity, which was a phyllosphere fungus with Quercus macrocarpa^(Jumpponen & Jones 2009)^ .

3 Can also be assigned to MG265969 uncultured fungus with more identity, which was foliar endophytes with Achnatherum lettermani ^(Kivlin^ *^et al.^* ^2019)^.


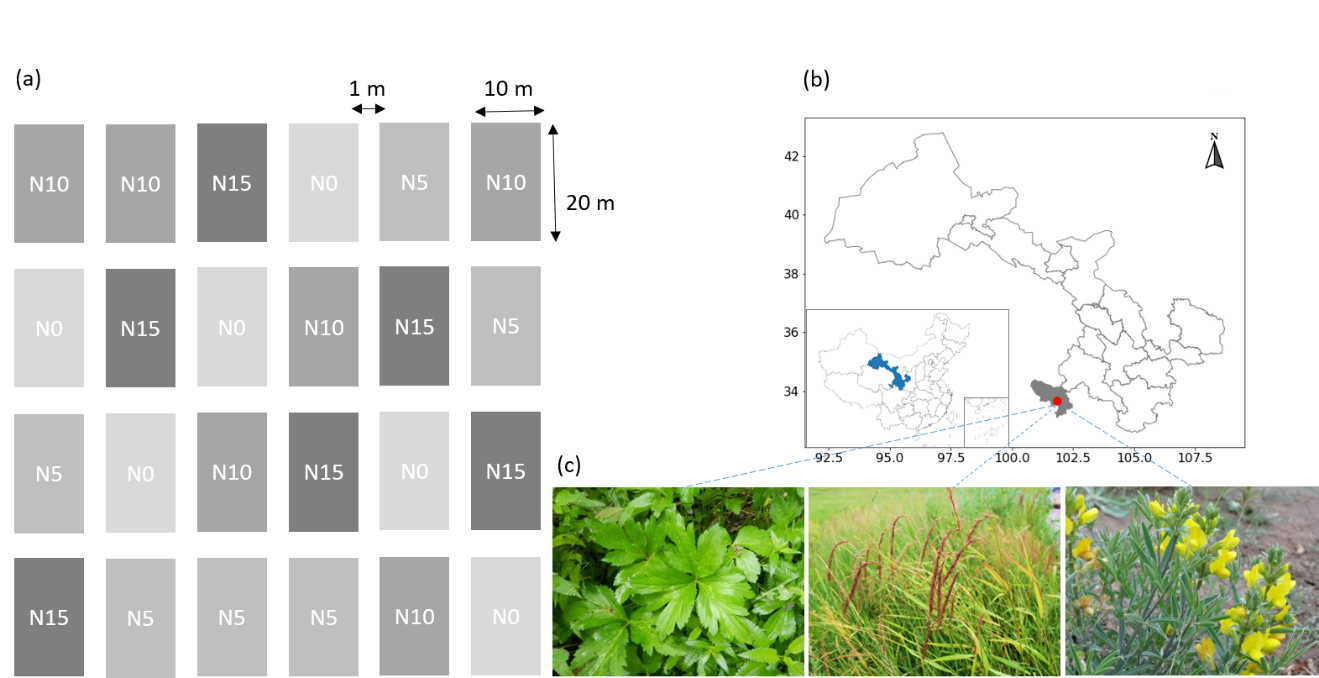


Fig S1. The experimental design (a), study site (b), and plant species of this study. (a) N0 (control), N5, N10 and N15 referring to 0, 5, 10 and 15 g N (NH4NO3) m-2 yr-1 treatments, respectively. (c) From left to right, *Anemone rivularis*, *Elymus nutans*, and *Thermopsis lanceolata.*


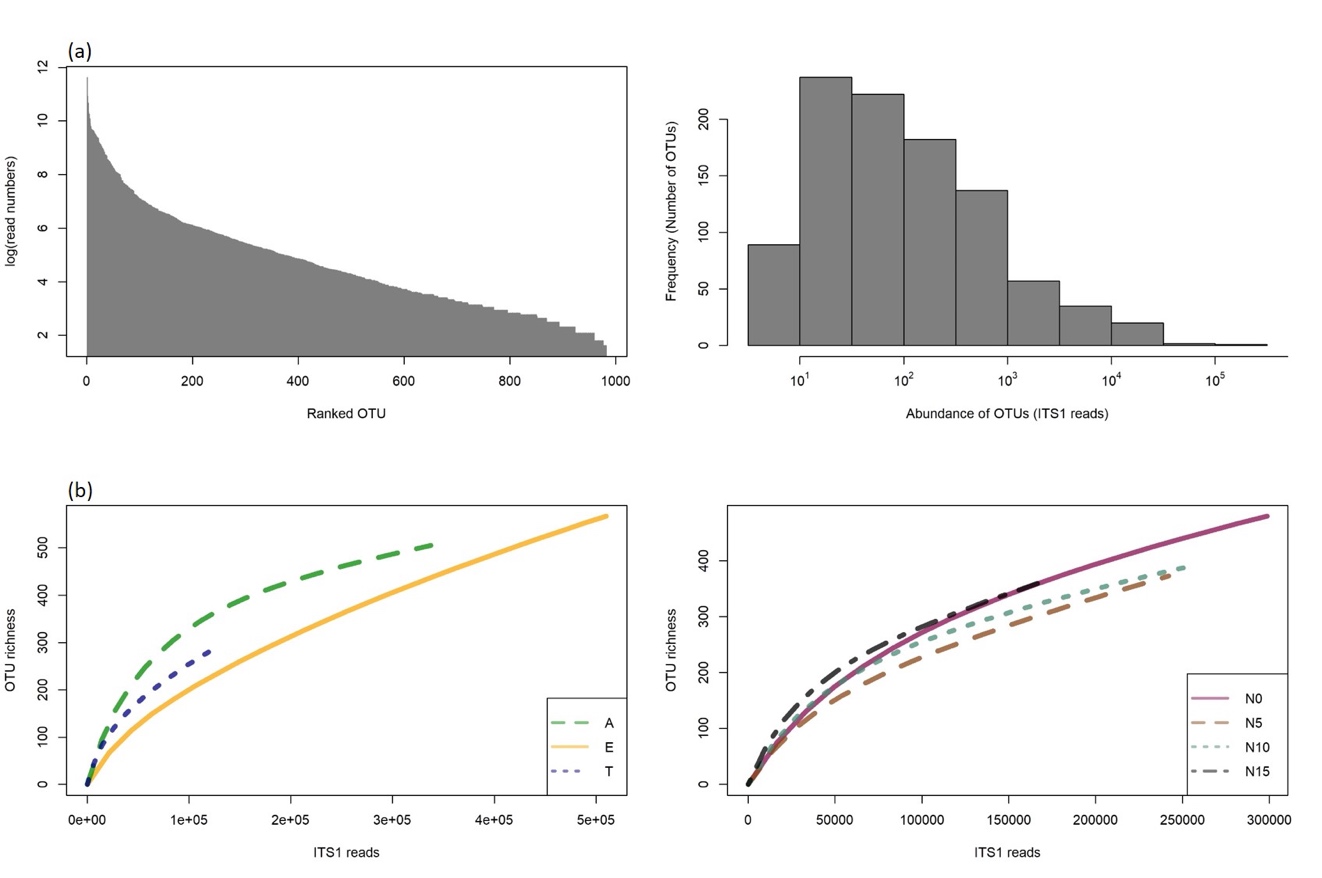


Fig. S2 Statistics of OTUs and read numbers obtained in this study (a) and rarefaction curves of observed OTU richness at the species and N addition levels (b). OTUs were designated based on 97% similarity of the ITS1 rDNA region.


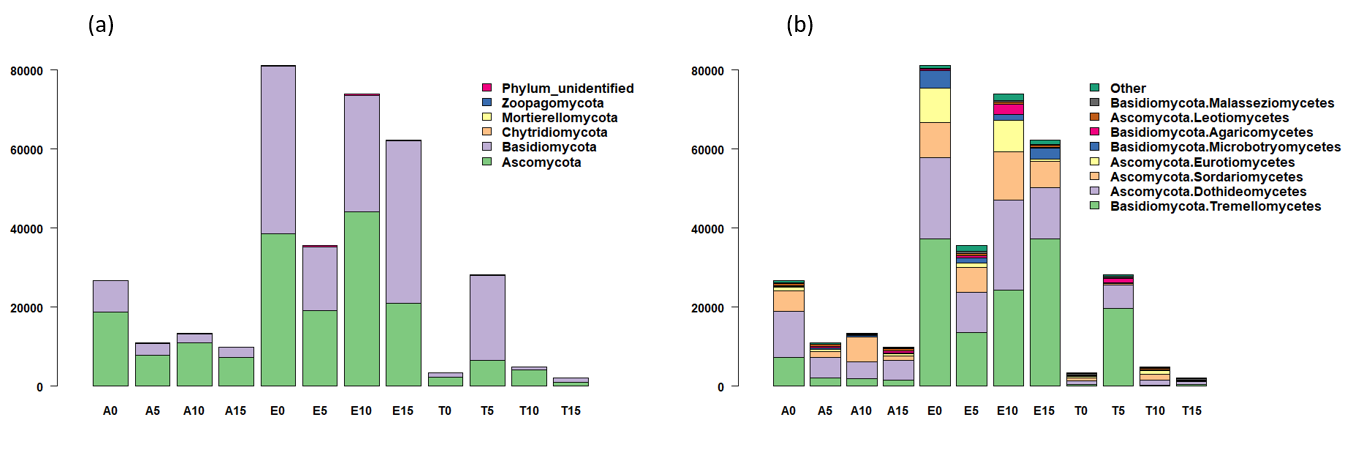


Fig. S3. Taxonomic composition, (a) phylum level, (b) class level, of the fungal communities recovered from the leaf interiors of three species along the nitrogen enrichment gradients in the study site. Bars show the frequency with which the different taxonomic groups were recovered. The fungal endophytes classes with relative abundances of < 0.1% were assigned to ‘Other’. A: Anemone rivularis, E: Elymus nutans, T: Thermopsis lanceolata. 0 (control), 5, 10 and 15 referring to 0, 5, 10 and 15 g N (NH_4_NO_3_) m-2 yr-1 treatments, respectively.


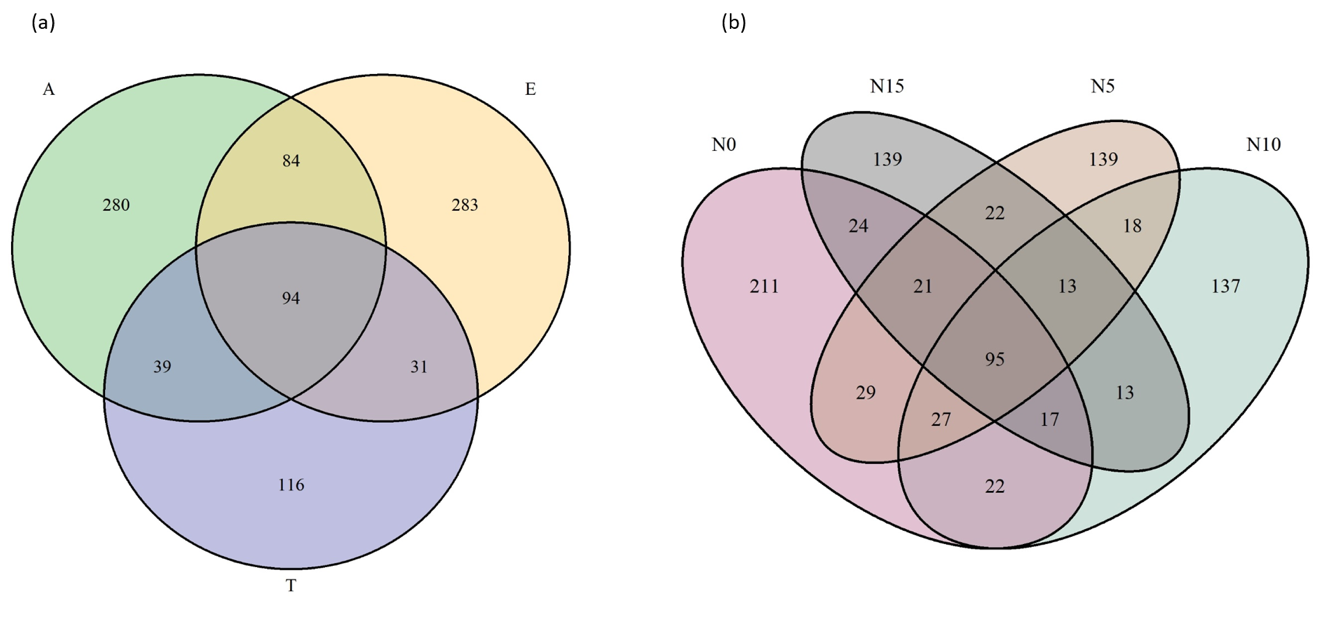


Fig. S4 Venn diagram showing the number of shared OTUs among the three hosts (a) and four N gradients (b). A: Anemone rivularis, E: Elymus nutans, T: Thermopsis lanceolata. N0, N5, N10 and N15 referring to 0, 5, 10 and 15 g N (NH_4_NO_3_) m-2 yr-1 treatments, respectively. *n* = 927 (rarefied data).


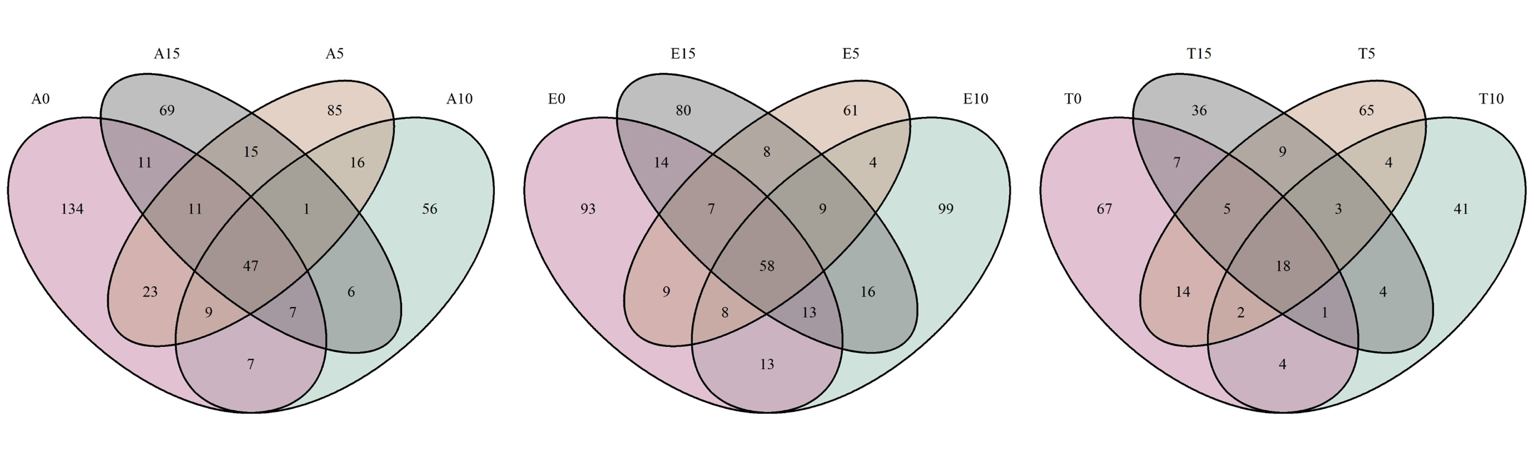


Fig. S5 Venn diagram showing the number of shared OTUs among four N gradients within each host. A: Anemone rivularis, E: Elymus nutans, T: Thermopsis lanceolata. 0, 5, 10 and 15 referring to 0, 5, 10 and 15 g N (NH_4_NO_3_) m-2 yr-1 treatments, respectively. *n* = 497, 492 and 280, respectively (rarefied data).

References

1.

Barge, E.G. (2019). Structure and Function of Foliar Fungal Communities of Populus trichocarpa Across its Native Range, Pacific Northwest, USA.

2.

Cordier, T., Robin, C., Capdevielle, X., Fabreguettes, O., Desprez-Loustau, M.L. & Vacher, C. (2012). The composition of phyllosphere fungal assemblages of European beech (Fagus sylvatica) varies significantly along an elevation gradient. *New Phytologist*, 196, 510-519.

3.

Golzar, H., Thomas, G., Jayasena, K.W., Wright, D., Wang, C. & Kehoe, M. (2019). Neoascochyta species cause leaf scorch on wheat in Australia. *Australas Plant Dis*, 14.

4.

Jumpponen, A. & Jones, K.L. (2009). Massively parallel 454 sequencing indicates hyperdiverse fungal communities in temperate Quercus macrocarpa phyllosphere. *New Phytologist*, 184, 438-448.

5.

Kivlin, S.N., Kazenel, M.R., Lynn, J.S., Taylor, D.L. & Rudgers, J.A. (2019). Plant Identity Influences Foliar Fungal Symbionts More Than Elevation in the Colorado Rocky Mountains. *Microb Ecol*, 78, 688-698.

6.

Kivlin, S.N. & Rudgers, J.A. (2019). Chapter 5 - Direct and indirect influences of warming on leaf endophytic fungi: A physiological and compositional approach. In: *Ecosystem Consequences of Soil Warming* (ed. Mohan, JE). Academic Press, pp. 125-140.

7.

Whitaker, B.K., Christian, N., Chai, Q. & Clay, K. (2020). Foliar fungal endophyte community structure is independent of phylogenetic relatedness in an Asteraceae common garden. *Ecology and Evolution*, 10, 13895-13912.

8.

Yeh, Y.-H. & Kirschner, R. (2019). Diversity of endophytic fungi of the coastal plant Vitex rotundifolia in Taiwan. *Microbes and environments*, 34, 59-63.
